# Multi-Omics Analyses Identify *ANLN* as a Prognostic Biomarker for Recurrence and Metastasis in Non-Small Cell Lung Cancer

**DOI:** 10.3390/genes17040461

**Published:** 2026-04-15

**Authors:** Haiwei Quan, Zhiguang Xu, Lizhen Huo, Zhibin Wang

**Affiliations:** 1 Department of Biomedical Engineering, Southern University of Science and Technology, Shenzhen 518055, China; 12333523@mail.sustech.edu.cn (H.Q.); 13260168199@163.com (L.H.); 2 Department of Biopharmaceutical Sciences, Faculty of Pharmaceutical Sciences, Shenzhen University of Advanced Technology, Shenzhen 518107, China; zg.xu2@siat.ac.cn; 3 Center for Cancer Immunotherapy, Institute of Biomedicine and Biotechnology, Shenzhen Institutes of Advanced Technology, Chinese Academy of Sciences, Shenzhen 518172, China

**Keywords:** *ANLN*, lung cancer, metastasis, single cell, epithelial cells

## Abstract

Background: Lung cancer recurrence and metastasis are major causes of cancer-related mortality, but the molecular determinants underlying these processes remain incompletely understood. This study aimed to identify key regulators of lung cancer progression through integrative analyses of bulk and single-cell transcriptomic datasets. Methods: Bulk transcriptomic and single-cell RNA sequencing data from multiple cohorts were integrated to identify genes associated with survival, recurrence, and metastasis. Tumor microenvironment features were further analyzed to prioritize core progression-related genes. *ANLN* was subsequently evaluated in independent single-cell datasets, followed by functional validation using CRISPR–Cas9-mediated gene knockout in lung cancer cells. Network-based drug prediction and molecular docking were performed to identify candidate compounds targeting *ANLN*-related programs. Results: *ANLN* was identified as a core progression-related gene associated with poor prognosis. *ANLN* was upregulated in recurrent and metastatic lung tumors and correlated with worse overall survival. Single-cell analyses showed that *ANLN* was predominantly expressed in epithelial and proliferating tumor cells and was associated with microenvironment remodeling, enhanced proliferative and migratory programs, and progression toward an invasive phenotype. These findings were validated in an independent single-cell dataset capturing the transition from in situ to invasive lung cancer. Functional experiments showed that *ANLN* deletion reduced proliferation and promoted apoptosis in lung cancer cells. Drug prediction and molecular docking identified several candidate compounds, among which Trifluridine and Monobenzone showed favorable binding potential and pro-apoptotic effects in lung cancer cells. Conclusions: *ANLN* is a potential regulator of lung cancer recurrence and metastasis and marks a conserved invasion-prone epithelial state. *ANLN*-associated pathways may represent potential therapeutic targets in lung cancer.

## 1. Introduction

Lung cancer remains the most diagnosed malignancy and the leading cause of cancer-related mortality globally [1]. Clinically, Lung cancer is broadly classified into non-small cell lung cancer (NSCLC) and small cell lung cancer (SCLC), with NSCLC accounting for approximately 85% of all cases [2]. Lung adenocarcinoma (LUAD) and lung squamous cell carcinoma (LUSC) are the two predominant histological subtypes of NSCLC. Although therapeutic strategies such as surgery, chemotherapy, radiotherapy, and combination regimens have achieved progress in controlling lung cancer progression [3], clinical outcomes for NSCLC patients remain suboptimal. Lung cancer is characterized by a low five-year survival rate and patients without recurrence beyond five years are typically considered cured [4]. Notably, approximately 20–40% of patients with stage I NSCLC still experience tumor recurrence after surgical resection [5,6,7], and late recurrence still occurs in approximately 8–11% of cases [8,9]. This highlights the urgent need to elucidate the mechanisms underlying disease progression and recurrence.

Cancer metastasis is one of the leading causes of cancer-related death [10,11]. It represents a highly complex, multistep process involving the coordinated activation of multiple biological pathways. This process includes the acquisition of invasive and migratory capabilities by tumor cells, detachment from the primary tumor site, survival during dissemination, and colonization of distant organs to form secondary tumors [12,13,14]. Cancer metastasis is governed by intricate biological processes, in which tumor cells undergo epithelial–mesenchymal transition, metabolic reprogramming, and escape from immune surveillance [15,16,17]. Despite substantial advances in elucidating metastasis-associated molecular mechanisms, the key regulatory factors that drive the transition of tumor cells from localized growth to metastatic dissemination remain incompletely understood.

Tumor recurrence and metastasis are complex events driven by multiple biological processes, including enhanced tumor cell proliferation, increased migratory capacity, and immune evasion [18]. Accumulating evidence has implicated genomic alterations and epigenetic dysregulation in the malignant progression of lung cancer [19,20,21]. Additionally, the tumor microenvironment (TME) plays a critical role in driving metastasis: for instance, enrichment of alveolar type II epithelial cells and tumor microenvironment remodeling characterized by macrophage polarization have been associated with malignant progression [22,23,24]. Recent studies have increasingly applied integrated single-cell, bulk, and even spatial transcriptomic approaches to investigate NSCLC heterogeneity, progression, and metastatic evolution. However, these efforts have largely focused on specific cellular compartments or immune subsets, and a unified framework linking clinical outcome, recurrence, metastasis, epithelial plasticity, and dynamic cell-state transitions at single-cell resolution remains insufficiently explored [25,26,27,28]. Such an integrated approach is essential to identify key regulators that govern the metastatic cascade and could serve as potential therapeutic targets. More broadly, the evolution of cancer therapy has increasingly shifted from nonspecific treatment modalities toward mechanism-based precision strategies, driven by advances in the understanding of cancer biology and molecular mechanisms [29]. In this context, identifying molecular regulators that not only correlate with clinical outcome but also functionally contribute to recurrence, metastasis, and epithelial plasticity has become particularly important. Such regulators are relevant not merely as biomarkers, but also as potential therapeutic nodes linking tumor biology to translational intervention.

Herein, we applied an integrative analytical strategy combining two survival cohorts (N = 83 and N = 176, respectively), a recurrence cohort (N = 119 recurrent and N = 152 non-recurrent samples), a metastasis cohort (N = 28 metastatic and N = 45 non-metastatic samples), a lymph-node single-cell RNA-seq dataset (N = 22 samples; n = 129,738 cells after quality control), and an independent single-cell validation dataset (N = 9 samples; n = 145,570 cells after quality control) to systematically investigate NSCLC progression. By integrating survival, recurrence, and metastasis analyses with co-expression network analysis, microenvironment inference, intercellular communication analysis, and pseudotime trajectory analysis, we characterized the links between candidate genes and epithelial plasticity, microenvironmental remodeling, and metastatic evolution. Through this approach, we identified *ANLN* as a key determinant of a highly plastic and invasion-prone epithelial state, providing new insight into the molecular mechanisms of NSCLC metastasis and highlighting *ANLN* as a potential therapeutic target for improving clinical outcomes.

## 2. Materials and Methods

### 2.1. Data Collection

To investigate key genes associated with lung cancer recurrence and metastasis, we retrieved publicly available datasets from the Gene Expression Omnibus (GEO): GSE41271, GSE166720, GSE277742, and GSE189357. Additionally, we collected two datasets related to overall survival (OS) in lung cancer patients: GSE157009 and GSE42127. After filtering, samples included those with recurrence (n = 119) or metastasis (n = 28), and non-recurrence (n = 152) or non-metastasis (n = 45) for downstream analyses. All datasets were processed and analyzed using R (v4.5.2). Differential gene expression was assessed using the limma package (v3.58.1), applying thresholds of |log_2_ FC| ≥ 0.3 with *p* < 0.05, and |log_2_ FC| ≥ 0.58 with *p* < 0.01 for the identification of recurrence or metastasis-associated genes.

### 2.2. Survival Analysis

Survival analysis was performed using the R packages survival (v3.5.7) and survminer (v0.4.9). A survival object was constructed via Surv, followed by fitting a Cox proportional-hazards model with coxph to estimate hazard ratio (HR) and *p*-value. Kaplan–Meier survival curves were then generated using survfit and visualized by ggsurvplot v0.5.2.

### 2.3. Immune Infiltration Analysis

Patients were stratified according to the expression of the key gene signature associated with lung cancer recurrence and metastasis, and their tumor transcriptome matrices were processed by xCell (v1.1.0) to estimate the relative abundance of diverse immune and stromal cell populations (cell-type enrichment scores) within each sample.

### 2.4. Enrichment Analysis

To explore the biological pathways involved in genes associated with lung cancer metastasis and recurrence, gene set enrichment analysis (GSEA) was performed on transcriptomic data [30]. An ordered gene list was generated by ranking all differentially expressed genes from high to low. This ranked list was then compared against the Hallmark, Gene Ontology (GO), and KEGG gene sets. The results of this analysis are used to identify key genetic functions and signaling pathways associated with lung cancer recurrence and metastasis.

### 2.5. CellChat Analysis

To compare intercellular communication between the *ANLN*-high and *ANLN*-low expression groups, the CellChat pipeline (v.1.6.1) was used to analyze cell–cell signaling interactions. A separate CellChat object was constructed for each group from the corresponding Seurat objects [31]. The human CellChatDB database was used as the reference. Communication probabilities were computed using the truncated mean method. Intercellular communication networks for each group were then inferred and aggregated using default parameters. To assess differences in cell–cell communication between the high and low *ANLN* expression groups, the total number of inferred interactions and the overall interaction strength were calculated and compared. Differential signaling pathways between the two groups were analyzed, and the relative contribution of specific signaling pathways was further evaluated.

### 2.6. scRNA-seq Analysis

Single-cell data from two independent datasets were processed separately using Seurat v5.3.12 [32] following a consistent workflow. Raw count matrices for each dataset were loaded individually, converted into Seurat objects, and merged within each dataset while retaining sample-level metadata. Quality control excluded cells with fewer than 200 or more than 10,000 detected genes, fewer than 500 UMI counts, or mitochondrial or hemoglobin gene fractions greater than 10%. After normalization, 3000 highly variable genes were identified, followed by data scaling and principal component analysis. To correct for inter-sample batch effects within each dataset, Harmony was applied using the original. ident as the batch variable [33]. The Harmony-corrected embeddings were then used for nearest-neighbor graph construction, clustering, and UMAP visualization [32,34]. Unless otherwise specified, clustering was performed using the first [35]. Harmony dimensions with a resolution of 0.8.

### 2.7. Gene-Signature Scoring

To evaluate malignant programs within epithelial cells at single-cell resolution, we defined four gene sets representing distinct biological processes: Proliferation, Motility, Immunity, and Metastasis. The Proliferation set was based on the term “GOBP mitotic nuclear division”, “GOBP cell division”, “KEGG cell cycle”, “GOBP epithelial cell proliferation” and “GOBP mitotic cytokinesis”, “GOBP DNA replication”; the Motility set was based on “GOBP extracellular matrix organization” and “GOBP cortical actin cytoskeleton organization”; the Immunity set utilized the “GOBP adaptive immune response” and “KEGG natural killer cell mediated cytotoxicity”; the Metastasis set aggregated these three gene sets. The AddModuleScore package was used to calculate scores for each cell and applied in downstream pseudotime, correlation, and functional analyses.

### 2.8. Pseudotime Analysis

To investigate dynamic state transitions, pseudotime trajectory analysis was conducted using Monocle-based approaches [36] in epithelial-cell and proliferating-cell subsets. For each analysis, normalized count matrices and corresponding cell metadata exported from Seurat were converted into trajectory analysis objects, followed by dimensionality reduction and trajectory graph learning. Cells were then ordered in pseudotime using the built-in trajectory ordering functions of the Monocle framework. Root states were determined computationally from the learned trajectory graph by the package’s internal ordering procedure, based on the topology of the inferred trajectory and the annotated identity of the analyzed cell subset. In the epithelial-cell analysis, epithelial-like tumor cells were considered to represent a relatively earlier state in tumor progression, providing the biological rationale for their use as the starting population [24,37]. Module scores representing metastasis, EMT, stemness, and proliferation-related programs were subsequently mapped along the pseudotime continuum to characterize coordinated transcriptional changes.

### 2.9. CRISPR–Cas9-Mediated ANLN Knockout

A lentivirus-mediated CRISPR–Cas9 system was used to delete *ANLN* in A549 cells using two independent sgRNAs. Two *ANLN*-targeting sgRNAs were designed based on the *ANLN* gene sequence and synthesized (BGI, Shenzhen, China). The oligonucleotides were annealed and cloned into BsmBI-digested lentiCRISPR v2 (Addgene, #52961, Watertown, MA, USA). A non-targeting sgRNA was used as the negative control. The sgRNA sequences (5′–3′) were as follows: sg1: TGCAGAGCAACGGCGCCGTT and sg2: TTGCAGAGCAACGGCGCCGT. Recombinant lentiCRISPR v2 plasmids (targeting and control) were transformed into DH5α competent cells (Beyotime, Shanghai, China) and cultured overnight at 37 °C and 180 rpm (~16 h) to allow plasmid amplification. Plasmids were then extracted using a miniprep kit (Novozan, Hangzhou, China) according to the manufacturer’s instructions. For lentiviral packaging, HEK293T cells (ATCC, CRL-3216, Manassas, VA, USA) were co-transfected with lentiCRISPR v2-sgRNA, psPAX2, and pMD2.G (Addgene) using polyethylenimine (PEI) (Beyotime). Viral supernatants were collected 48 h after transfection, filtered, and used as lentiviral particles. A549 cells (ATCC, CCL-185, Manassas, VA, USA) were subsequently infected with the lentiviral supernatant for 24 h, followed by routine culture. The knockout efficiency was evaluated by extracting total RNA and quantifying *ANLN* mRNA levels using RT-qPCR.

### 2.10. Cell Culture

A549, NCI-H1975 (ATCC, CRL-5908, Manassas, VA, USA), and HEK293T cells were cultured in complete medium under standard conditions (37 °C, 5% CO_2_). A549 and H1975 cells were used for *ANLN* knockout experiments, whereas HEK293T cells were used for lentiviral packaging.

### 2.11. RT-qPCR

Total RNA was extracted using TRIzol. For cultured cells, the medium was removed, cells were washed with PBS, and lysed directly in TRIzol (1 mL/well of a 6-well plate). Chloroform was added (200 μL), samples were mixed, incubated, and centrifuged at 12,000× *g*, 4 °C for 15 min. The aqueous phase was precipitated with isopropanol, washed with 75% ethanol, air-dried, and dissolved in DEPC-treated water. RNA concentration was measured by UV spectrophotometry. cDNA was synthesized using a reverse transcription kit (Vazyme, R223, Nanjing, China) with gDNA removal. qPCR was performed using 2× ChamQ SYBR qPCR Master Mix (Vazyme, Q711).

Primers (5′–3′):

- *ANLN*-F: ATTGGAAGCAACTGCAGCCT
- *ANLN*-R: GGAGTTGCCGAGGCATTTGA
- *CDH1*-F: CATCACTGGCCAAGGAGCTG
- *CDH1*-R: CGTTGGATGACACAGCGTGA
- *SNAI1*-F: AGCGAGCTGCAGGACTCTAA
- *SNAI1*-R: GCCAGGACAGAGTCCCAGAT
- *VIM*-F: CGGGAGAAATTGCAGGAGGAG
- *VIM*-R: CAAGGTCAAGACGTGCCAGAG
- *CDH2*-F: TGCGGTACAGTGTAACTGGG
- *CDH2*-R: GAAACCGGGCTATCTGCTCG

### 2.12. CFSE-Based Proliferation Assay

A549 cells in logarithmic growth phase were trypsinized (0.25% trypsin–EDTA), neutralized with complete medium, collected, and washed twice with cold PBS to remove serum. Cells were resuspended in PBS and labeled with CFSE (MedChemExpress, HY-D0938, Monmouth Junction, NJ, USA) at a final concentration of 5 μM and incubated for 15 min at 37 °C in the dark, mixing every 5 min. Labeling was quenched by adding 3× volume of complete medium with 10% FBS, followed by incubation for 10 min. Cells were washed twice (1400 rpm, 4 °C, 7 min) to remove excess dye, counted, and seeded at 6 × 10^4^ cells/well in 24-well plates. After 48 h, CFSE fluorescence intensity was measured by flow cytometry to evaluate proliferation.

### 2.13. Apoptosis Assay by Flow Cytometry (Annexin V-FITC/PI)

Cells were harvested, washed twice with cold PBS, and resuspended in Annexin V binding buffer to 1 × 10^6^ cells/mL. Aliquots (100 μL) were stained with Annexin V-FITC (5 μL) and PI (5 μL) (BD Pharmingen, #556547, San Diego, CA, USA) for 15 min at room temperature in the dark, followed by addition of 400 μL binding buffer. Samples were analyzed immediately using NovoCyte Advanteon VBR (Agilent, Santa Clara, CA, USA), collecting 10,000 events per sample, with three technical replicates. Data were analyzed using NovoExpress (1.6.2.0) to calculate apoptotic fractions.

### 2.14. Cell Counting

Cells were trypsinized in 6-well plates, neutralized, and resuspended. Cell numbers were determined using a hemocytometer. Only cells in the lower and left boundary grids were counted. Cell density was calculated as:

Cell density (cells/mL) = (total cells/number of large squares) × 10^4^.

### 2.15. Molecular Docking

Molecular docking was performed to evaluate the potential interactions between candidate compounds and *ANLN*. The three-dimensional structure of *ANLN* was first predicted using Boltz2. Because the ligand-binding pocket of *ANLN* was unknown, binding pockets were first predicted to define the docking region. The predicted pockets were then used for subsequent docking refinement. The *ANLN* structure was used as the receptor and candidate compounds were used as ligands. Docking was performed using AutoDock Vina (v1.2.7), and binding affinities were calculated as binding energies (kcal/mol). For each compound, the conformation with the lowest binding energy was selected as the representative docking result. Candidate compounds identified based on *ANLN* co-expression network analysis are listed in Table 1, and their molecular docking results with *ANLN* are presented in Table 2.

### 2.16. WGCNA Analysis

Weighted gene co-expression network analysis (WGCNA) was performed in R (v4.3.2) using the WGCNA package (v1.73) [38]. Given the distinct data characteristics of the metastasis and recurrence cohorts, separate gene prefiltering strategies were applied prior to network construction to balance biological signal retention and network robustness. In the metastasis cohort, genes were retained after filtering based on expression abundance and differential-expression criteria, yielding approximately 5000 genes for WGCNA. In the recurrence cohort, the top 3000 most variable genes were selected to reduce noise and focus the analysis on the dominant transcriptional heterogeneity. The soft-thresholding power was determined using the pickSoftThreshold v1.74 function. The selected power was 6 for the metastasis cohort and 10 for the recurrence cohort. Co-expression networks were then constructed using the blockwiseModules function with TOMType = "unsigned", minModuleSize = 30, reassignThreshold = 0, and mergeCutHeight = 0.25. Module eigengenes were calculated and correlated with the corresponding clinical traits to identify modules associated with metastasis or recurrence.

### 2.17. Pan-Cancer Expression Analysis Using TIMER2.0

Pan-cancer expression analysis of *ANLN* was performed using the TIMER2.0 web server. Differential expression of *ANLN* between tumor and adjacent normal tissues across multiple cancer types was evaluated based on The Cancer Genome Atlas (TCGA) datasets integrated within the platform. Expression differences were analyzed using the built-in “Gene_DE” module, which applies the Wilcoxon rank-sum test for comparisons between tumor and normal samples. Statistical significance was defined as *p* < 0.05 unless otherwise specified. The resulting expression profiles were visualized using box plots generated by the TIMER2.0 platform [39].

### 2.18. Network-Based Drug Prediction Using Enrichr

An *ANLN*-centered co-expression gene set was constructed by selecting the top 100 genes most positively correlated with *ANLN* expression from the gene co-expression matrix. This gene set was uploaded to Enrichr for drug perturbation enrichment analysis. Candidate compounds were ranked by enrichment statistics (*p* value, adjusted *p* value, odds ratio, combined score), and the top-ranked compounds are summarized in Table 1.

## 3. Results

### 3.1. An Integrated Analysis of Survival, Recurrence, and Metastasis Identifies a Core Gene Set Critical for Lung Cancer Progression

**Figure 1:**
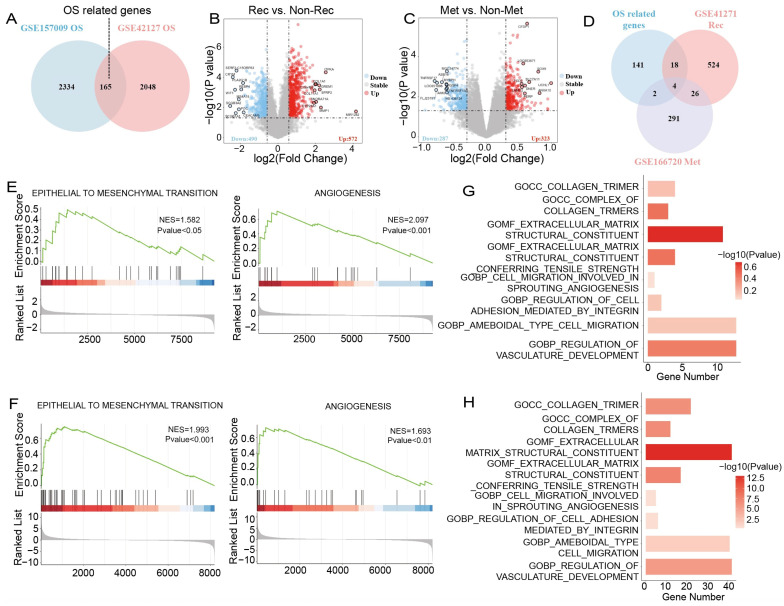
An integrated analysis of survival, recurrence, and metastasis identifies a core gene set critical for lung cancer progression. (**A**) Venn diagram illustrating the overlap of OS-associated genes from GSE157009 and GSE42127, with 165 genes shared between the two cohorts. (**B**) Volcano plot showing differentially expressed genes between recurrence and non-recurrence samples in GSE157009 (572 upregulated). (**C**) Volcano plot showing differentially expressed genes between metastatic and non-metastatic samples in GSE42127 (323 upregulated). (**D**) Venn diagram integrating OS, recurrence, and metastasis-associated genes, revealing four overlapping core genes. (**E**) GSEA of recurrence-associated upregulated genes demonstrates enrichment of EMT and Angiogenesis pathways; red indicates the upregulated end of the ranked gene list, and blue indicates the downregulated end. (**F**) GSEA of metastasis-associated upregulated genes showing similar enrichment of EMT and Angiogenesis; red indicates the upregulated end of the ranked gene list, and blue indicates the downregulated end. (**G**,**H**) GO enrichment analyses showing ECM remodeling, adhesion, migration, and angiogenesis pathways enriched in both recurrence (**G**) and metastasis (**H**) gene sets.

Survival analysis of the GSE157009 and GSE42127 datasets identified 2499 and 2213 genes associated with overall survival (OS), with 165 genes overlapping between the two cohorts (Figure 1A), which represents a cross-dataset consensus survival-associated signature. Differential expression analysis comparing the Recurrence (Rec) group with the Non-Recurrence (Non-Rec) group revealed 572 genes that were significantly upregulated in the Rec group (Figure 1B). In contrast, 323 genes were upregulated in the Metastasis (Met) group relative to the corresponding control group (Figure 1C), indicating substantial transcriptomic reprogramming associated with both progression phenotypes. The Venn diagram integrating 165 survival-related genes with the 572 recurrence-upregulated and 323 metastasis-upregulated genes identified four shared genes—*ANLN*, *TPX2*, *CDCA8*, and *RAD54L* (Figure 1D), suggesting that these genes may act as key regulators simultaneously linked to recurrence, metastasis and reduced overall survival. 

Further analysis of the upregulated genes using GSEA revealed significant enrichment of the EMT and Angiogenesis pathways in both the Rec (Figure 1E) and Met groups (Figure 1F). These pathways are known biological drivers of tumor invasion, facilitating cancer cell migration and dissemination. In addition, GO analyses indicated that extracellular matrix remodeling, collagen assembly, regulation of cell adhesion and migration, and vasculature development were all upregulated in the Rec and Met groups (Figure 1G,H). These processes form the essential microenvironmental basis that promotes tumor invasion, which is consistent with the malignant biological characteristics of recurrence and metastasis observed in these samples.

### 3.2. WGCNA and xCell Analyses Identify ANLN as a Key Biomarker Associated with Tumor Metastasis and Recurrence

**Figure 2:**
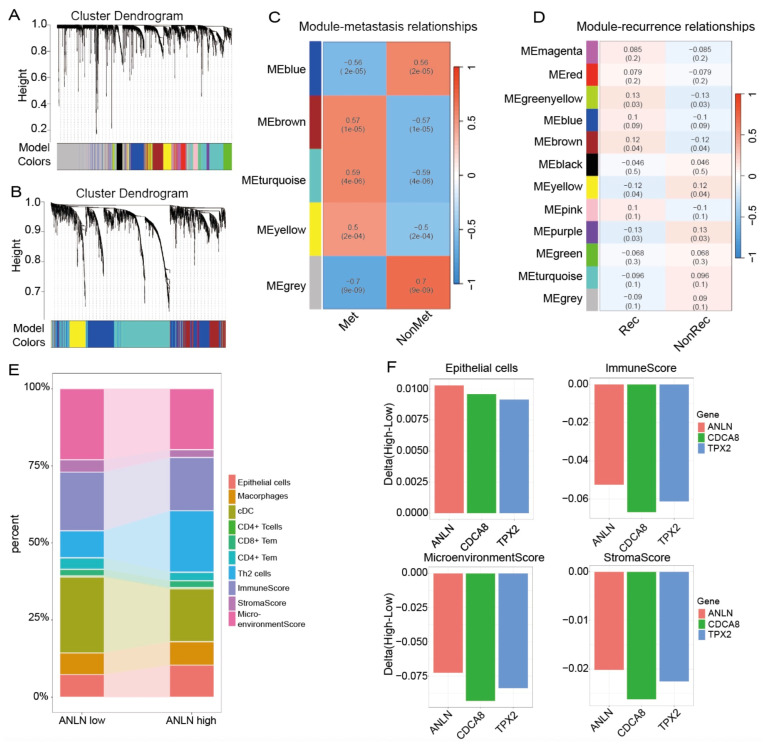
(**A**) Hierarchical clustering dendrogram of the metastasis cohort showing the co-expression module structure identified by WGCNA; the color bar below the dendrogram indicates module assignment, with different colors representing distinct co-expression modules and grey indicating unassigned genes. (**B**) Hierarchical clustering dendrogram of the recurrence cohort showing the co-expression module structure identified by WGCNA; the color bar below the dendrogram indicates module assignment, with different colors representing distinct co-expression modules and grey indicating unassigned genes. (**C**) Correlation analysis between co-expression modules and metastatic status. The MEturquoise module shows the strongest positive correlation with metastasis and includes *ANLN*, *TPX2*, and *CDCA8*. (**D**) Correlation analysis between co-expression modules and recurrence status. The MEbrown module shows a significant positive correlation with recurrence and likewise includes *ANLN*, *TPX2*, and *CDCA8*. (**E**) xCell-based comparison of tumor microenvironment composition between the *ANLN*-low and *ANLN*-high groups in the recurrence cohort. The *ANLN*-high group shows increased epithelial cell enrichment together with relative reductions in immune and stromal-related components. (**F**) Comparative analysis of xCell-derived changes between high and low expression groups for *ANLN*, *TPX2*, and *CDCA8*. Among the three candidate genes, the *ANLN*-high group shows the most prominent increase in epithelial cell enrichment, whereas all three high-expression groups exhibit reduced ImmuneScore, MicroenvironmentScore, and StromaScore.

Given that the integrated analysis above identified four candidate core genes associated with survival, recurrence, and metastasis, we next applied WGCNA and xCell analyses to further evaluate their network context and associations with tumor microenvironment remodeling. In the metastasis and recurrence cohorts, genes with similar expression patterns were grouped into distinct co-expression modules by hierarchical clustering. As shown in Figure 2A,B, genes were grouped into multiple color-coded modules according to their expression patterns, providing the basis for subsequent module–trait association analysis.

Module–trait correlation analysis further showed that, in the metastasis cohort, the MEturquoise module was most strongly and positively associated with metastatic status, whereas in the recurrence cohort, the MEbrown module showed a significant positive correlation with recurrence, as shown in Figure 2C,D. Among the four candidate core genes identified in the previous step, *ANLN*, *TPX2*, and *CDCA8* were simultaneously assigned to both the metastasis-associated MEturquoise module and the recurrence-associated MEbrown module, indicating that these three genes belong to a shared co-expression network linked to both recurrence and metastasis. This result further narrowed the range of candidate driver genes and provided a basis for selecting the most representative gene for downstream analysis.

To further compare the association between these candidate genes and tumor microenvironment remodeling, xCell analysis was performed in the recurrence cohort, and samples were stratified into high and low expression groups according to the expression levels of *ANLN*, *TPX2*, and *CDCA8*, respectively. As shown in Figure 2E, the *ANLN* high group exhibited increased epithelial cell enrichment, accompanied by decreases in ImmuneScore, StromaScore, and MicroenvironmentScore, suggesting that tumors with high *ANLN* expression tend to display a microenvironmental state characterized by relative epithelial enrichment and reduced immune and stromal components.

A comparative evaluation of the magnitude of microenvironmental changes across the three candidate genes further showed that, although high *TPX2* and high *CDCA8* expression were also associated with reductions in immune, stromal, and overall microenvironment scores, the *ANLN* high group showed the most prominent increase in epithelial cell enrichment, as shown in Figure 2F. Given the important role of epithelial-related programs in lung cancer recurrence and metastasis, the microenvironmental pattern associated with *ANLN* was more consistent with the biological features of malignant progression. Therefore, compared with *TPX2* and *CDCA8*, *ANLN* showed a stronger association between intrinsic tumor progression programs and tumor microenvironment remodeling, and was thus prioritized as the representative key gene for subsequent in-depth investigation.

### 3.3. Independent Evidence Identifies ANLN as a Key Progression-Related Gene in Lung Cancer

Expression and prognostic analyses further establish *ANLN* as a key progression-associated gene in lung cancer.

**Figure 3:**
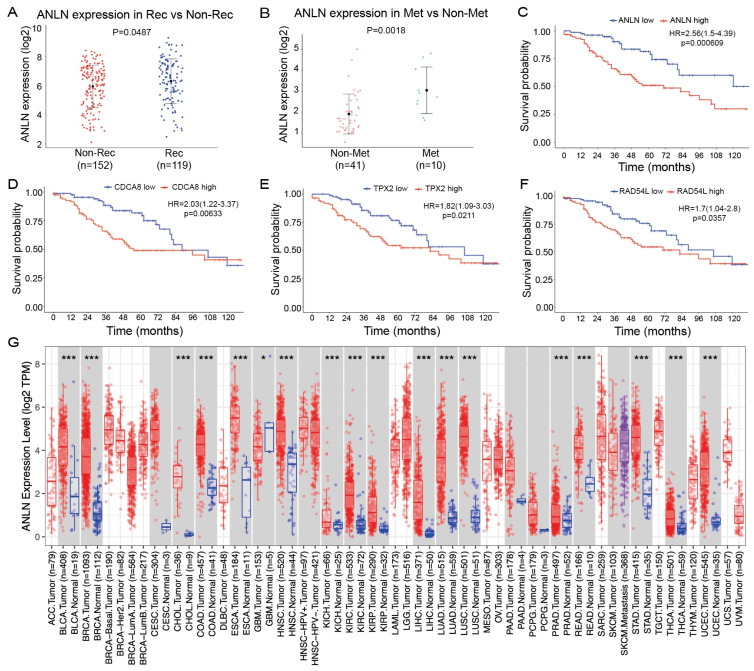
(**A**) Comparison of *ANLN* expression between recurrent (Rec) and non-recurrent (Non-Rec) samples. *ANLN* expression is significantly increased in the Rec group (*p* = 0.0487). (**B**) Comparison of *ANLN* expression between metastatic (Met) and non-metastatic (Non-Met) samples. *ANLN* expression is significantly elevated in the Met group (*p* = 0.0018). (**C**–**F**) Kaplan–Meier survival analyses in the GSE42127 cohort showing overall survival differences between high- and low-expression groups of *ANLN* (**C**), CDCA8 (**D**), *TPX2* (**E**), and *RAD54L* (**F**). High expression of each gene is associated with poorer survival, with *ANLN* showing the strongest prognostic impact. (**G**) Pan-cancer expression analysis of *ANLN* based on the TIMER2.0 database. *ANLN* is significantly upregulated in multiple tumor types compared with corresponding normal tissues (most comparisons *p* < 0.001), indicating widespread dysregulation across cancers. Statistical significance is indicated as follows: *, *** *p* < 0.001.

Comparative expression analysis revealed that *ANLN* was significantly upregulated in recurrent tumors compared with non-recurrent tumors (*p* = 0.0487; Figure 3A), and was likewise markedly elevated in metastatic samples relative to non-metastatic samples (*p* = 0.0018; Figure 3B). Kaplan–Meier survival analyses in the GSE42127 cohort further demonstrated that high expression of *ANLN*, *CDCA8*, *TPX2*, and *RAD54L* was consistently associated with poorer overall survival (Figure 3C–F). Among these candidates, *ANLN* showed the strongest prognostic impact, with a hazard ratio (HR) of 2.56 (95% CI: 1.54–4.39, *p* = 0.00069), exceeding those observed for *CDCA8* (HR = 2.03), *TPX2* (HR = 1.82), and *RAD54L* (HR = 1.70). These results suggest that, although all four genes are linked to unfavorable prognosis, *ANLN* exhibits superior risk stratification capability and may represent the most clinically relevant biomarker within this gene set.

To further evaluate the broader relevance of *ANLN*, pan-cancer expression analysis based on the TIMER2.0 database was performed. As shown in Figure 3G, *ANLN* was significantly upregulated in multiple tumor types, including lung adenocarcinoma and lung squamous cell carcinoma, with most comparisons reaching strong statistical significance (*p* < 0.001). This widespread overexpression pattern suggests that *ANLN* activation is not restricted to lung cancer but represents a conserved molecular feature across diverse malignancies.

Taken together, the WGCNA results linking *ANLN* to recurrence and metastasis-associated gene modules, the xCell analysis indicating that high *ANLN* expression is associated with epithelial enrichment and progression-related microenvironment remodeling, and the expression and prognostic analyses collectively identify *ANLN* as the most representative key gene for lung cancer progression and justify its prioritization for subsequent study.

### 3.4. ANLN-High Epithelial Cells Are Markedly Enriched in Lymph-Node Metastatic Lesions and Exhibit Extensive Cell Type-Specific Transcriptional Reprogramming

**Figure 4:**
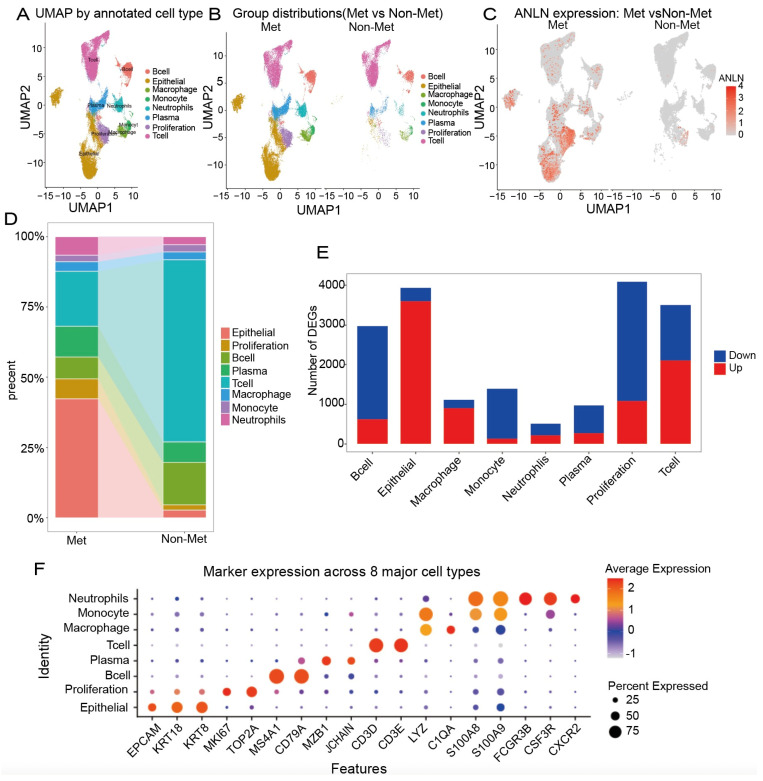
(**A**) UMAP visualization of 145,570 integrated lymph-node cells from metastatic (Met) and non-metastatic (Non-Met) lung cancer samples, identifying eight major cell types: Epithelial, Proliferation, T cells, B cells, Macrophages, Monocytes, Neutrophils, and Plasma cells. (**B**) Comparison of cell-type composition shows a marked increase in epithelial and proliferating tumor cells in metastatic lymph nodes, whereas immune lineages (T cells, myeloid cells) are proportionally enriched in non-metastatic samples. (**C**) *ANLN* expression map indicates that *ANLN* is predominantly expressed in epithelial and proliferative compartments, with substantially stronger and broader expression in metastatic lesions. (**D**) Quantitative analysis of population shifts further demonstrates epithelial and proliferation expansion and immune depletion in metastatic samples. (**E**) Differential expression analysis across eight cell types reveals that epithelial cells harbor the largest number of DEGs between Met and Non-Met samples, highlighting epithelial transcriptional rewiring as a major driver of lymph-node metastasis. (**F**) Dot-plot of canonical markers validates the identities of the eight annotated cell populations supporting downstream analyses.

To further dissect the role of *ANLN* in lung cancer metastasis, we analyzed the GSE277742 dataset, which contains single-cell RNA sequencing data (scRNA-seq) from lymph nodes of 18 lung cancer patients with lymph node metastasis (Met group) and 4 patients without metastasis (Non-Met group). After quality control, normalization, dataset integration, and principal component analysis (PCA), a total of 145,570 cells were retained for downstream analyses Annotation of the integrated single-cell dataset identified eight major cell populations, including Epithelial cells (*EPCAM*, *KRT8*, *KRT18*), Proliferation cells (*MKI67*, *TOP2A*), T cells (*CD3D*, *CD3E*), B cells (*MS4A1*, *CD79A*), Macrophages (*C1QA*), Monocytes (*FCGR3B*, *CSF3R*, *CXCR2*), Neutrophils (*S100A8*, *S100A9*), and Plasma cells (*MZB1*, *JCHAIN*) (Figure 4A,F).

Examination of *ANLN* expression across different clinical groups (Figure 4C) revealed that *ANLN* signals were predominantly localized to epithelial and proliferation-related clusters, with markedly stronger and broader expression in the Met group. This suggests that *ANLN*-high epithelial cells constitute the major tumor cell population within metastatic lymph nodes. Quantitative comparison of cellular composition between the Met and Non-Met groups (Figure 4B,D) further showed that the Met group exhibited a substantial increase in epithelial and proliferating cells, whereas immune populations, including T cells and myeloid cells, were relatively enriched in the Non-Met group. These findings indicate a transition toward an epithelial-dominated cellular architecture in metastatic lymph nodes. We next performed differential expression analysis within each of the eight cell populations (Figure 4E). The proliferation and epithelial compartments showed the largest and second largest number of differentially expressed genes, underscoring the pivotal role of epithelial cells in the metastatic progression of lung cancer.

Collectively, these results demonstrate that lung cancer lymph node metastasis is characterized by the accumulation of *ANLN*-high epithelial tumor cells and extensive transcriptional and compositional remodeling across multiple cell lineages, providing single-cell evidence for the central role of *ANLN* in metastatic dissemination.

### 3.5. High ANLN Expression Enhances Metastatic Pathways in Epithelial Cells During Lymph Node Metastasis of Lung Cancer

**Figure 5:**
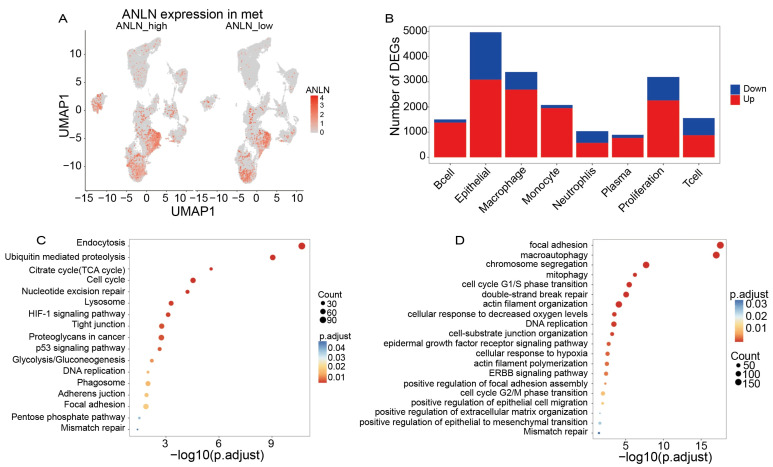
(**A**) UMAP visualization shows markedly elevated *ANLN* expression in epithelial and proliferating cells in the *ANLN*-high group. (**B**) Differential expression analysis reveals the largest DEG changes in epithelial, proliferative, and macrophage populations, with additional remodeling in T cells and monocytes. (**C**) KEGG enrichment highlights activation of cell cycle, DNA replication, p53 signaling, and metabolic pathways in *ANLN*-high tumors. (**D**) GO analysis identifies enrichment of adhesion, migration, cytoskeletal remodeling, DNA repair, and EMT-related processes.

To further elucidate the functional role of *ANLN* within metastatic lymph nodes, we stratified the 18 metastatic samples into *ANLN*-high (N = 9) and *ANLN*-low (N = 9) groups based on *ANLN* expression levels and compared their single-cell characteristics. UMAP visualization (Figure 5A) showed markedly elevated *ANLN* expression in the high expression group, with signals predominantly localized to epithelial and proliferation-associated cell populations.

Building on these observations, we next examined differential gene expression across the major single-cell compartments between the *ANLN*-high and *ANLN*-low groups (Figure 5B). The largest numbers of DEGs were detected in the epithelial, proliferation, and macrophage clusters, indicating that high *ANLN* expression not only alters transcriptional programs within tumor epithelial cells but is also associated with substantial transcriptional changes in immune cells—particularly macrophages. Notable DEG alterations were also observed in T-cell and monocyte populations, suggesting that *ANLN*-associated remodeling exerts broad, multi-lineage effects across the metastatic tumor ecosystem.

To investigate *ANLN*-related regulatory pathways, KEGG and GO enrichment analyses were performed using DEGs derived from the comparison between *ANLN*-high and *ANLN*-low groups (Figure 5C,D). KEGG analysis revealed significant enrichment in canonical oncogenic processes, including Cell cycle progression, Chromosome segregation, DNA replication, p53 signaling, Glycolysis/Gluconeogenesis, and Ubiquitin-mediated proteolysis. GO analysis further highlighted enrichment in local adhesion, cell migration, double-strand break repair, epithelial cell migration, epithelial–mesenchymal transition (EMT), and extracellular matrix organization, which are key biological programs implicated in tumor invasion and dissemination.

Collectively, these findings demonstrate that in lung cancer lymph node metastasis, high *ANLN* expression defines a highly proliferative and strongly migratory epithelial cell population, accompanied by widespread transcriptional alterations across multiple cell types. These results suggest that *ANLN* may promote metastatic progression by reprogramming tumor epithelial cells and reshaping their surrounding microenvironment.

### 3.6. CellChat Analysis Reveals Enhanced Intercellular Communication Strength and Selective Activation of Metastasis-Associated Signaling Pathways in ANLN-High Tumors

**Figure 6:**
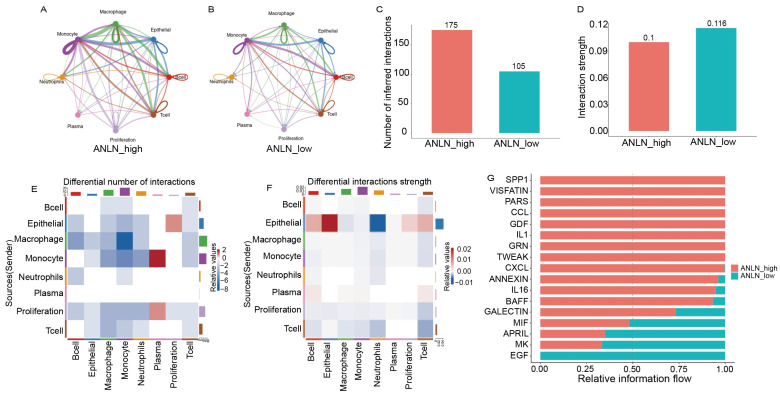
(**A**,**B**) CellChat-inferred intercellular communication networks in *ANLN*-high and *ANLN*-low tumors, respectively. Different colors denote different cell populations, and the line colors correspond to the source cell type of each interaction. (**C**,**D**) Quantification of the total number of inferred interactions (**C**) and overall interaction strength (**D**) in the *ANLN*-high and *ANLN*-low groups. (**E**,**F**) Differential heatmaps of interaction number (**E**) and interaction strength (**F**). Heatmap colors represent relative changes in interaction number or interaction strength, whereas the colored annotations along the top and right margins indicate the corresponding cell populations using the same color scheme as in subfigures (**A**,**B**). (**G**) Relative information flow analysis reveals that pro-metastatic pathways—including SPP1, VISFATIN, PARs, CCL, GDF, IL-1, GRN, TWEAK, CXCL, ANNEXIN, IL-16, BAFF, and GALECTIN—are strongly elevated in *ANLN*-high tumors, reflecting enhanced inflammation, migration, immune modulation, and ECM remodeling.

To systematically investigate intercellular communication patterns associated with *ANLN* expression, we systematically compared the intercellular communication patterns between the *ANLN*-high and *ANLN*-low groups using CellChat analysis. Figure 6A–D illustrates the global communication networks among the eight major cell populations in the two groups. The *ANLN*-high group exhibited a weaker overall interaction strength but a markedly greater number of intercellular interactions than the *ANLN*-low group. This pattern suggests that *ANLN*-high metastatic lesions harbor a more broadly connected but less concentrated communication network. In other words, although several tumor- and metastasis-related pathways, including SPP1, CCL, CXCL, IL-1, TWEAK, and GALECTIN, showed increased relative information flow in the *ANLN*-high group, the global interaction strength remained slightly lower because communication was distributed across a larger number of cell–cell pairs, many of which were likely of modest intensity rather than highly weighted interactions. Such a pattern is consistent with the concept that metastatic progression is supported by broad bidirectional crosstalk among tumor, immune, and stromal compartments rather than by a single dominant signaling axis [40]. Given that *ANLN* has been implicated in tumor-cell proliferation, migration, and invasive behavior [41], this distributed communication architecture may reflect a more plastic and extensively remodeled metastatic microenvironment. In particular, macrophage-associated communication may contribute to this phenotype, as tumor-associated macrophages are known to promote EMT, extracellular matrix remodeling, and tumor-cell migration, including in NSCLC [42,43].

At the cell-type level, we assessed the differences in interaction patterns between the two groups (Figure 6E,F). Figure 6E depicts the heatmap of differential interaction counts, and Figure 6F shows the corresponding differences in interaction strength. The results revealed that the incoming and outgoing signaling of epithelial, proliferation, and macrophage populations was substantially elevated in the *ANLN*-high group, suggesting that *ANLN* mediates coordinated remodeling across multiple cell types within the metastatic microenvironment.

We further compared the relative information flow of signaling pathways between *ANLN*-high and *ANLN*-low groups (Figure 6G). Pathways including SPP1, VISFATIN, PARs, CCL, GDF, IL-1, GRN, TWEAK, CXCL, ANNEXIN, IL-16, BAFF, and GALECTIN were markedly enhanced in the *ANLN*-high group. These ligand–receptor axes are broadly implicated in inflammatory amplification, cellular migration, immune modulation, and extracellular matrix (ECM) remodeling, consistent with highly invasive biological programs associated with metastasis. Similar immune-associated signaling networks have increasingly been recognized as potential therapeutic targets in cancer, particularly in the context of cancer immunotherapy, further suggesting that *ANLN*-associated communication pathways may intersect with immunoregulatory mechanisms during tumor progression [44].

### 3.7. High ANLN Expression Is Associated with Enhanced Epithelial EMT and Migration-Related Programs

**Figure 7:**
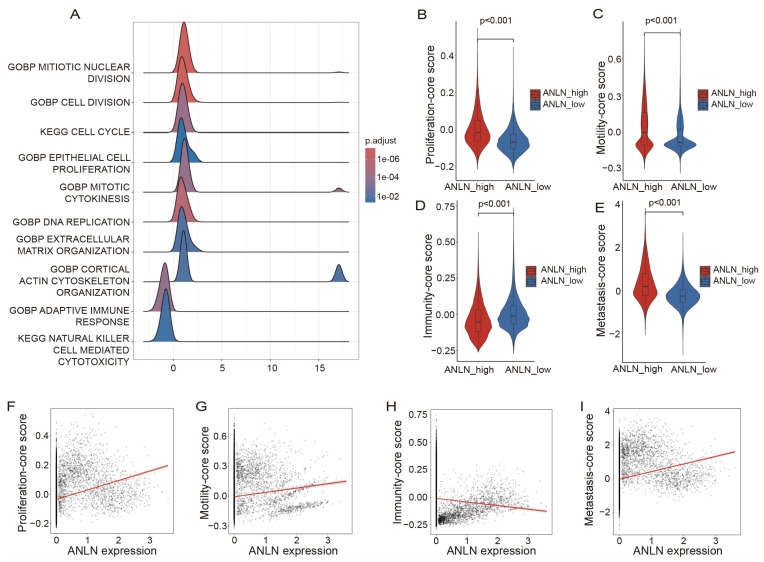
(**A**) GSEA plot comparing *ANLN*+ vs *ANLN*− epithelial cells, showing enrichment of Proliferation, Motility, and Immune-related pathways used to construct three module scores. (**B**) Proliferation score is significantly higher in *ANLN* high samples. (**C**) Motility score is significantly elevated in *ANLN* high samples. (**D**) Immunity score is significantly lower in *ANLN* high samples. (**E**) Metastasis score is markedly increased in *ANLN* high tumors. (**F**) Single-cell correlation showing *ANLN* expression positively correlates with proliferation score (Spearman ρ = 0.186, *p* < 0.01). (**G**) *ANLN* expression positively correlates with motility score (Spearman ρ = 0.118, *p* < 0.01). (**H**) *ANLN* expression negatively correlates with immunity score (Spearman ρ = −0.176, *p* < 0.01). (**I**) *ANLN* expression positively correlates with the composite metastasis score (Spearman ρ = 0.215, *p* < 0.01). The red line indicates the fitted trend line.

To further elucidate the functional programs associated with *ANLN* in metastatic epithelial cells, we first stratified epithelial cells from the lymph node single-cell dataset into *ANLN*-expressing (*ANLN*^+^) and *ANLN*-non-expressing (*ANLN*^−^) populations. Differential expression analysis was performed between these two groups, followed by gene set enrichment analysis (GSEA). The results revealed that *ANLN*^+^ epithelial cells were significantly enriched for pathways related to cell proliferation, cell motility, and immune regulation compared with *ANLN*^−^ epithelial cells (Figure 7A).

Based on the GSEA results, three functional module scores were constructed. Proliferation score was calculated using genes derived from the top six proliferation- and cell cycle-related pathways, Motility score was computed from genes associated with migration and cytoskeletal remodeling, and Immunity score was derived from immune-related pathways. Using these scores, metastatic samples were stratified into *ANLN*-high and *ANLN*-low groups at the sample level, and functional differences between the two groups were systematically evaluated.

Compared with *ANLN*-low tumors, epithelial cells from *ANLN*-high tumors exhibited significantly elevated proliferation scores (Figure 7B) and motility scores (Figure 7C), whereas immunity scores were markedly reduced (Figure 7D). To capture the integrated metastatic potential, a composite metastasis score was calculated by combining the proliferation and motility scores with the immunity score. Notably, the composite metastasis score was significantly higher in *ANLN*-high tumors (Figure 7E), indicating a globally enhanced pro-metastatic transcriptional state.

To further assess these associations at a continuous single-cell level, correlation analyses were performed across the epithelial cells from metastatic samples. *ANLN* expression showed significant positive correlations with the proliferation score (Figure 7F) and motility score (Figure 7G), while displaying a negative correlation with the immunity score (Figure 7H). In addition, *ANLN* expression was positively correlated with the composite metastasis score (Figure 7I).

Collectively, these findings demonstrate that high *ANLN* expression is tightly linked to coordinated activation of proliferation and migration-related programs and suppression of immune-associated transcriptional activity in epithelial cells, supporting a central role for *ANLN* in promoting epithelial plasticity and metastatic progression in lung cancer.

### 3.8. Pseudotime Trajectory Analysis of AT2-Derived Epithelial Cells Revealed That ANLN Defines a Critical Transitional State

**Figure 8:**
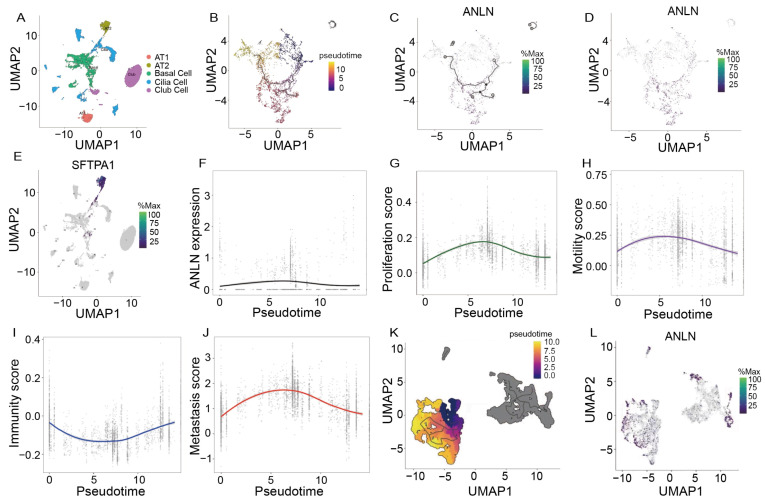
(**A**) UMAP of epithelial subclusters (AT1, AT2, basal, cilia, club) from metastatic lymph nodes. (**B**) Monocle-inferred pseudotime trajectory of epithelial cells. (**C**,**D**) *ANLN* expression overlaid on the trajectory and UMAP, showing enrichment at an intermediate pseudotime region. (**E**) Feature plot of the AT2 marker SFTPA1. (**F**) *ANLN* expression dynamics along pseudotime. (**G**–**J**) Program score changes along pseudotime: Proliferation (**G**), Motility (**H**), Immunity (**I**), and Metastasis score (**J**). (**K**) Pseudotime trajectory reconstructed within proliferation cells. (**L**) UMAP of *ANLN* expression overlaid on the proliferating-lineage trajectory.

To further elucidate the dynamic role of *ANLN* during metastatic progression, we performed subclustering and pseudotime trajectory analysis on epithelial cells from metastatic lymph nodes. UMAP visualization identified five major epithelial subpopulations, including AT1, AT2, basal, ciliated, and club cells (Figure 8A). Expression of the AT2 marker *SFTPA1* was predominantly enriched in the AT2 cluster (Figure 8E). AT2 is the origin of lung cancer cells [22,23,24], so it is crucial to analyze the development of this subgroup.

Monocle-based trajectory inference revealed a continuous temporal progression of AT2 cells’ state along the principal lineage (Figure 8B). Mapping *ANLN* expression onto this trajectory demonstrated that *ANLN* was not maximally expressed at the initial or terminal stages, but instead showed prominent enrichment at an intermediate region of the trajectory (Figure 8C,D). Quantitative analysis further confirmed that *ANLN* expression increased in a stage-dependent manner and peaked during the intermediate pseudotime window (Figure 8F), suggesting that *ANLN* marks a critical transitional phase during epithelial progression rather than a static lineage endpoint.

To characterize the functional features of this *ANLN*-high transitional state, we examined the dynamic changes in multiple programmatic scores along pseudotime. The proliferation score increased markedly at the intermediate stage (Figure 8G), indicating activation of cell-cycle-related programs. In parallel, the motility score also peaked in the mid-trajectory region (Figure 8H), reflecting enhanced cytoskeletal remodeling and migratory capacity. In contrast, the immunity score exhibited a decreasing trend during the intermediate phase (Figure 8I), suggesting attenuation of immune-associated programs. Integration of these features into the composite metastasis score revealed a pronounced peak at the same intermediate pseudotime window (Figure 8J), closely mirroring the *ANLN* expression pattern and defining a state characterized by high proliferative and migratory potential coupled with reduced immune-related features.

To exclude the possibility that this pattern merely reflects a generic proliferative phenotype, we further constructed a separate pseudotime trajectory within the proliferation cells belonging to the eight subtypes identified in cancer metastasis components (Figure 8K). Notably, *ANLN* expression remained selectively enriched at specific mid-to-late stages of this trajectory (Figure 8L), rather than being uniformly elevated across all proliferating cells, indicating that *ANLN* marks a more advanced, invasion-prone functional state.

Collectively, these suggested a noteworthy pseudotime model in which *ANLN* marked an intermediate epithelial state during metastatic evolution. While the inferred trajectory provided a plausible model of metastatic progression, its biological significance remains to be further validated, as the proposed transitional state was derived primarily from single-cell transcriptomic inference rather than direct functional evidence. Thus, *ANLN* may mark a putative transition toward a more invasive phenotype rather than a definitively established metastatic intermediate state. Future validation could include isolating *ANLN*-high and *ANLN*-low epithelial populations based on expression patterns, or establishing inducible *ANLN* expression models, followed by Matrigel invasion assays or organoid or spheroid invasion models to compare their invasive potential. Such experiments would provide functional support for the inferred transitional phenotype. More broadly, the identification of *ANLN* as a candidate driver of metastatic progression did not arise from a single analytical layer, but from the convergence of survival-associated, recurrence-associated, metastasis-associated, microenvironment-related, and pseudotime-associated signals across multiple datasets. This integrative pattern suggests that *ANLN* may represent not merely an isolated marker, but part of a broader regulatory architecture underlying aggressive tumor phenotypes. In this regard, our findings align with the growing view in cancer systems biology that multi-omics and multi-level computational integration can help uncover complex oncogenic determinants that may not be evident from individual datasets alone [45].

### 3.9. Independent Single-Cell Validation Confirms Enrichment of ANLN-High Invasive Epithelial Programs During Lung Cancer Progression

**Figure 9:**
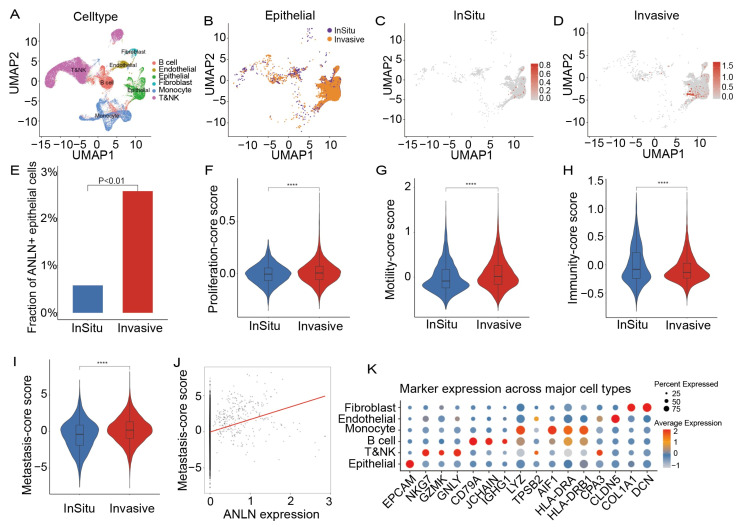
(**A**) UMAP of GSE189357 showing eight major cell types annotated by canonical markers. (**B**) UMAP of epithelial cells colored by stage (in situ vs invasive). (**C**,**D**) Feature plots of *ANLN* expression in epithelial cells from in situ (**C**) and invasive (**D**) tumors. (**E**) Fraction of *ANLN*-positive epithelial cells in in situ and invasive tumors (*p* < 0.01). (**F**–**H**) Violin plots comparing Proliferation (**F**), Motility (**G**), and Immunity (**H**) scores in epithelial cells between groups (*p* < 0.0001). (**I**) Violin plot of the composite metastasis score in epithelial cells (*p* < 0.0001). (**J**) Correlation between *ANLN* expression and metastasis score across epithelial cells (ρ = 0.243, *p* < 0.01). The red line indicates the fitted trend line. (**K**) Dotplot of canonical marker expression validating cell type annotation. Asterisks indicate statistical significance as follows: **** *p* < 0.0001.

To independently validate the association between *ANLN* expression and invasive epithelial programs, we analyzed the single-cell RNA-seq dataset GSE189357, which profiles the transition from in situ lung cancer to invasive lung cancer. After standard preprocessing and clustering, cells were annotated into eight major populations based on canonical markers, including epithelial cells, T/NK cells, B cells, monocytes, endothelial cells, fibroblasts, and others (Figure 9A,K).

We focused on epithelial cells and compared their distribution between in situ and invasive tumors (Figure 9B). Feature plots show sparse expression of *ANLN* in situ lesions, whereas invasive tumors exhibit stronger and more widespread *ANLN* expression within epithelial clusters (Figure 9C,D). Quantitative analysis further demonstrated that the fraction of *ANLN*-positive epithelial cells was significantly higher in invasive tumors than in situ tumors (*p* < 0.01; Figure 9E), indicating enrichment of *ANLN*-high epithelial cells during invasive progression.

To validate the functional scoring framework we defined, we applied the same Proliferation, Motility, Immunity, and Metastasis scores to epithelial cells in the GSE189357 dataset. Using these predefined gene sets, epithelial cells from invasive tumors exhibited significantly higher Proliferation and Motility scores with reduced Immunity scores, compared with those from in situ tumors (Figure 9F–H). Consistent with the coordinated behavior of these gene sets observed previously, the Metastasis score, integrating Proliferative, Migratory, and Immune scores, was markedly elevated in the invasive group (Figure 9I). Moreover, correlation analysis across all epithelial cells demonstrated a significant positive association between *ANLN* expression and the metastasis score (Figure 9J), confirming that the *ANLN*-linked metastatic program we identified is reproducibly captured in an independent single-cell dataset, reinforcing the role of *ANLN* as a marker and potential driver of invasive epithelial states during lung cancer progression.

### 3.10. CRISPR–Cas9-Mediated Deletion of ANLN Suppresses Proliferation and Promotes Apoptosis in Lung Cancer Cells

**Figure 10:**
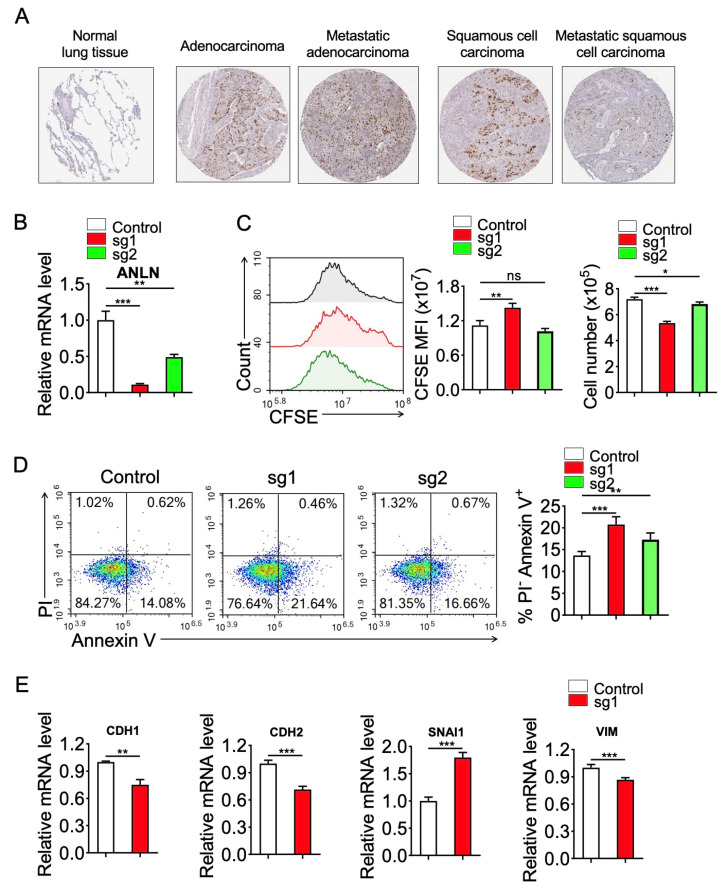
(**A**) Immunohistochemistry images from the Human Protein Atlas showing *ANLN* protein expression in normal lung tissue and different lung cancer tissues, including lung adenocarcinoma, metastatic adenocarcinoma, squamous cell carcinoma, and metastatic squamous carcinoma. (**B**) RT-qPCR analysis showing *ANLN* mRNA levels in control and *ANLN* knockout A549 cells generated using two independent sgRNAs (sg1 and sg2). (**C**) CFSE-based flow cytometry analysis of cell proliferation. Left: representative CFSE fluorescence histograms for control, sg1, and sg2 groups. Middle: quantification of CFSE mean fluorescence intensity (MFI). Right: total cell number measured after culture. (**D**) Flow cytometry analysis of apoptosis using Annexin V-FITC/PI staining. Left: representative apoptosis plots. Right: quantification of apoptotic cell fractions in control and *ANLN* knockout cells. (**E**) RT-qPCR analysis of EMT-related markers (*CDH1*, *CDH2*, *SNAI1*, and *VIM*) in control and *ANLN* knockout (sg1) A549 cells, showing decreased *CDH1*, *CDH2*, and *VIM* and increased *SNAI1* expression. Statistical significance is indicated as follows: ns, not significant; * *p* < 0.05; ** *p* < 0.01; *** *p* < 0.001.

Based on the integrative transcriptomic and single-cell analyses described above, we subsequently performed experimental validation to examine the functional role of *ANLN* in lung cancer cells. We used a lentivirus-mediated CRISPR–Cas9 system to delete *ANLN* in A549 lung adenocarcinoma cells using two independent sgRNAs (sg1 and sg2). In addition, we examined immunohistochemistry data from the Human Protein Atlas and found that *ANLN* protein expression was minimal in normal lung tissue but markedly elevated in lung adenocarcinoma, metastatic adenocarcinoma, squamous cell carcinoma, and metastatic squamous carcinoma tissues (Figure 10A), supporting the clinical relevance of *ANLN* upregulation in lung cancer.

We confirmed efficient *ANLN* knockout by RT-qPCR analysis, which demonstrated a substantial reduction in *ANLN* mRNA levels in both sg1 and sg2 groups compared with the control group (Figure 10B). To determine the effect of *ANLN* depletion on cell proliferation, we performed CFSE-based flow cytometry assays. Representative CFSE histograms showed a rightward shift in fluorescence intensity following *ANLN* knockout (Figure 10C), indicating reduced proliferation capacity. Quantitative analysis further confirmed that cells expressing sg1 exhibited significantly increased CFSE mean fluorescence intensity (MFI), consistent with slower cell division, whereas sg2 displayed a comparable but less pronounced trend. Consistently, we observed that the total cell numbers were significantly reduced in the *ANLN*-deficient groups relative to control cells.

**Figure A1:**
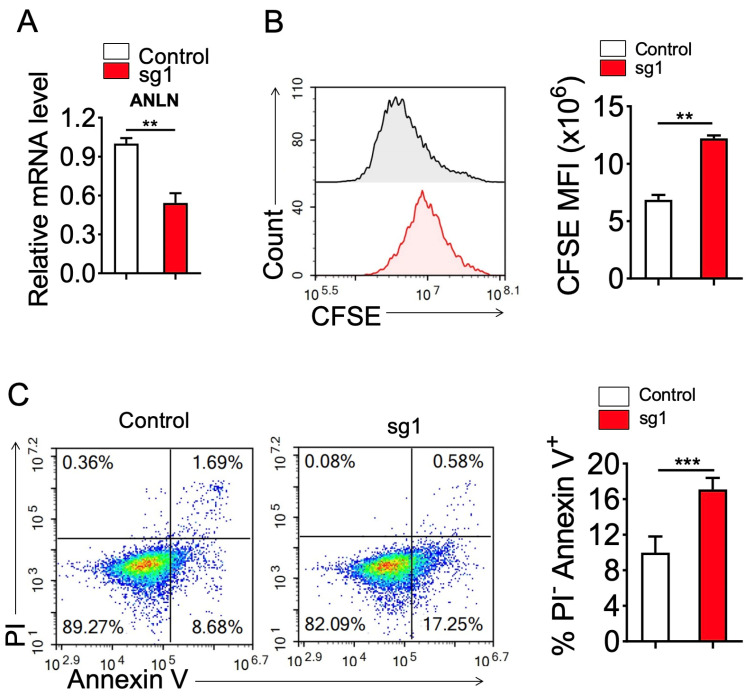
(**A**) RT-qPCR analysis of *ANLN* expression in control and sg1 groups, confirming efficient knockdown (*p* < 0.01). (**B**) CFSE-based flow cytometry analysis of cell proliferation. Left: representative CFSE fluorescence histograms; right: quantification of mean fluorescence intensity (MFI), indicating reduced proliferation in *ANLN*-knockdown cells (*p* < 0.01). (**C**) Annexin V–FITC/PI staining followed by flow cytometry analysis of apoptosis. Left: representative dot plots; right: quantification of apoptotic cells (% Annexin V+), showing increased apoptosis in *ANLN*-knockdown cells (*p* < 0.001). Statistical significance is indicated as follows: ** *p* < 0.01; *** *p* < 0.001.

We further assessed the effect of *ANLN* loss on cell survival by Annexin V–FITC/PI-based flow cytometry analysis. Representative dot plots revealed an increased proportion of apoptotic cells in both sg1 and sg2 groups compared with control cells (Figure 10D). Quantification of Annexin V^+^/PI^+^ populations further confirmed that *ANLN* knockout significantly increased apoptosis rates, indicating that *ANLN* contributes to tumor cell survival. Notably, additional validation experiments reproduced the inhibitory effects of *ANLN* depletion on proliferation and the pro-apoptotic phenotype (Appendix A, Figure A1), supporting the robustness of these observations.

To examine whether *ANLN* contributes to invasion-related transcriptional programs, we assessed the expression of key epithelial–mesenchymal transition (EMT) markers by RT-qPCR. *CDH1* (E-cadherin) is a canonical epithelial marker, whereas *CDH2* (N-cadherin) and *VIM* (*VIM*entin) are representative mesenchymal markers, and *SNAI1* is a core EMT-inducing transcription factor [46,47,48]. As shown in Figure 10E, *ANLN* knockout led to decreased expression of *CDH1* (*p* < 0.01), *CDH2* (*p* < 0.001), and *VIM* (*p* < 0.001), together with significantly increased *SNAI1* expression (*p* < 0.01). Combined with the concurrent suppression of proliferation and enhancement of apoptosis, these findings indicate that *ANLN* is required not only for sustaining tumor cell growth and survival but also for maintaining EMT-related transcriptional plasticity. Together with the multi-omics analyses linking *ANLN* to proliferative, migratory, and microenvironmental features, these results support *ANLN* as a functional regulator of malignant progression in lung cancer rather than merely a passive biomarker.

### 3.11. Small-Molecule Drug Prediction, Molecular Docking Analysis, and Functional Validation Based on the ANLN Co-Expression Network

Based on the multi-omics analyses and functional experiments described above, we next applied a network-based strategy to identify small-molecule compounds potentially associated with the *ANLN*-related molecular program in lung cancer.

We first constructed an *ANLN*-centered co-expression network to capture the broader transcriptional module associated with *ANLN* activity. Using gene co-expression analysis, we identified the top 100 genes that were most strongly correlated with *ANLN* expression and used this gene set as input for drug perturbation enrichment analysis in the Enrichr platform. The analysis identified several small-molecule compounds significantly associated with the *ANLN* co-expression gene set. The top predicted compounds ranked by statistical significance, odds ratio, and combined score are summarized in Table 1. Among these candidates, Trifluridine and Monobenzone ranked among the most significant compounds, suggesting that these drugs may influence *ANLN*-related transcriptional programs and potentially interfere with lung cancer progression.

We next evaluated the potential interaction between the candidate compounds and *ANLN* at the structural level using molecular docking. We performed rigid-receptor docking using AutoDock Vina, which searches multiple spatial conformations and calculates the corresponding binding free energies to estimate the interaction strength between each compound and the *ANLN* protein. The docking results of all candidate compounds are summarized in Table 1, and the representative docking scores of the top compounds are shown in Table 2. Among the tested molecules, Etoposide showed the lowest predicted binding energy with *ANLN*, suggesting the strongest docking interaction. However, as Etoposide is already a clinically used anticancer drug, we focused on additional candidate compounds for further validation. Trifluridine and Monobenzone, which ranked among the top predicted compounds, displayed favorable docking scores with minimum binding energies of approximately −7.5 kcal/mol and −7.0 kcal/mol, respectively, and were therefore selected for subsequent structural analysis and functional experiments.

**Figure 11:**
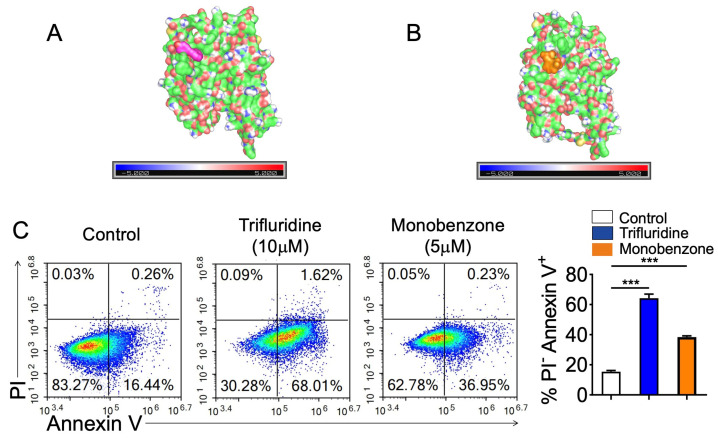
(**A**) Molecular docking model showing the predicted binding mode of Trifluridine with the *ANLN* protein. The ligand molecule is shown occupying a surface pocket of *ANLN*, suggesting a potential interaction interface. (**B**) Molecular docking model showing the predicted binding mode of Monobenzone with the *ANLN* protein. The compound is located within a surface groove of the *ANLN* structure, indicating a feasible binding configuration. (**C**) Flow cytometry analysis of apoptosis in A549 cells treated with candidate compounds. Left: representative Annexin V–FITC/PI staining plots for control, Trifluridine (10 μM), and Monobenzone (5 μM) groups. Right: quantification of apoptotic cell fractions. Both compounds significantly increased apoptosis compared with the control group, with Trifluridine showing the strongest pro-apoptotic effect (*** *p* < 0.001).

We further visualized the optimal docking conformations to examine the spatial interaction between the compounds and *ANLN*. As shown in Figure 11A, Trifluridine was predicted to insert into a structural pocket on the *ANLN* protein surface and form a stable binding conformation with surrounding residues. The ligand was positioned near a region associated with protein interaction and functional regulation, suggesting that the compound may influence *ANLN* activity by affecting its structural stability or interaction capacity. Similarly, Monobenzone was predicted to occupy a compatible binding site on the *ANLN* protein surface and form a stable docking configuration (Figure 11B). Although its binding energy was slightly weaker than that of Trifluridine, the docking model still demonstrated favorable structural complementarity without obvious steric conflicts.

Based on the docking results, we selected Trifluridine and Monobenzone for subsequent functional validation in lung cancer cells. We treated A549 lung cancer cells with Trifluridine (10 μM) or Monobenzone (5 μM) and assessed apoptosis using Annexin V-FITC/PI double staining followed by flow cytometry. Representative flow cytometry plots are shown in Figure 11C. In the control group, most cells were in the viable quadrant with a low proportion of apoptotic cells. In contrast, treatment with Trifluridine markedly increased the proportion of Annexin V-positive cells, indicating a substantial elevation of apoptotic populations. Monobenzone treatment also increased the apoptotic fraction compared with the control group, although the magnitude of the effect was weaker than that observed for Trifluridine.

Quantitative analysis further confirmed that Trifluridine treatment significantly increased the proportion of apoptotic cells, whereas Monobenzone induced a moderate but still significant increase (Figure 11C, right panel). The difference between the Trifluridine group and the control group reached a high level of statistical significance (*** *p* < 0.001). These results demonstrate that both compounds are capable of reducing lung cancer cell viability and promoting apoptosis.

These findings indicate that the network-based prediction strategy successfully identified candidate compounds targeting *ANLN*-associated transcriptional programs. Molecular docking analysis further supported the potential direct interaction between *ANLN* and the selected compounds, and functional experiments demonstrated that these molecules could induce apoptosis in lung cancer cells. These results provide additional evidence that *ANLN* represents a potentially druggable target in lung cancer and that Trifluridine and Monobenzone may serve as candidate therapeutic compounds.

## 4. Discussion

In recent years, the rapid development of multi-omics analyses has greatly advanced our understanding of tumor progression mechanisms and prognostic evaluation. In this study, by integrating multi-cohort transcriptomic data with single-cell analyses, we revealed the critical role of *ANLN* in lung cancer recurrence and metastasis. *ANLN* encodes an actin-binding protein that plays an essential role in cell growth, migration, and cytoskeletal dynamics [30]. Although *ANLN* has been reported to be associated with cell proliferation in multiple tumor types [31], its precise biological function and role in lung cancer metastasis remain insufficiently characterized. Herein, we performed an integrated analysis combining survival, recurrence, and metastasis phenotypes to explore the potential regulatory role of *ANLN* in malignant lung cancer progression.

We found that *ANLN* expression was consistently elevated in both recurrent and metastatic samples. In addition, high *ANLN* expression was significantly associated with poor overall survival. This poor overall survival indicates that *ANLN* may contribute to the formation of an aggressive tumor phenotype.

Based on our results, it seems that the profound impact of *ANLN* on the TME is through increased epithelial cell proportions and relative depletion of immune and stromal components. Our data further indicate the influence of *ANLN* on the TME is through predominant enrichment in epithelial and proliferating cell populations, which are accompanied by marked alterations in cellular composition and transcriptional programs. CellChat analysis further showed that *ANLN*-high tumors exhibited more complex intercellular communication networks and selectively activated multiple signaling pathways related to inflammatory regulation, cell migration, and extracellular matrix remodeling, suggesting that *ANLN* may promote metastatic progression by reshaping intercellular communication patterns. Consistently, subsequent GSEA and GO functional enrichment analyses revealed that genes associated with high *ANLN* expression were significantly enriched in pathways related to cell cycle regulation, epithelial–mesenchymal transition (EMT), cell migration, and extracellular matrix remodeling. These processes are widely recognized as fundamental molecular mechanisms underlying tumor metastasis, further supporting the hypothesis that *ANLN* plays a promotive role in lung cancer progression.

By integrating functional scoring analyses with pseudotime trajectory inference, we further demonstrated that *ANLN* is not merely a proliferation marker but represents a critical intermediate cellular state during metastatic progression. Specifically, *ANLN* marks the transition of AT2-derived epithelial cells toward a highly invasive phenotype characterized by enhanced proliferative and migratory capacities together with suppression of immune-related programs. Importantly, the functional relevance of *ANLN* was further supported by experimental validation. CRISPR–Cas9-mediated deletion of *ANLN* significantly reduced the proliferative capacity of lung cancer cells and promoted apoptosis, indicating that *ANLN* contributes to tumor cell survival and growth. These findings provide functional evidence supporting the role of *ANLN* in sustaining malignant cellular programs during lung cancer progression.

Our findings are broadly consistent with previous reports showing that *ANLN* is associated with aggressive tumor behavior. In lung adenocarcinoma, *ANLN* overexpression has been reported to promote migration, invasion, and EMT-like changes, supporting its role in metastatic progression [49]. More broadly, prior studies have suggested that *ANLN* functions not only as a prognostic biomarker but also as a regulator of tumor cell proliferation and migration across multiple cancer types [43]. Compared with these earlier studies, our work extends the current understanding by integrating survival, recurrence, metastasis, tumor microenvironment remodeling, and single-cell state transition analyses, thereby linking *ANLN* to a broader progression-associated epithelial program in NSCLC. Nevertheless, several limitations should be acknowledged. First, much of our mechanistic inference is based on transcriptomic and computational analyses rather than direct pathway-level experimentation. Second, although our functional experiments support a role for *ANLN* in proliferation, apoptosis, and EMT-related transcriptional plasticity, the precise molecular mechanisms by which *ANLN* regulates metastatic progression remain to be further defined. Third, the predicted *ANLN*-associated therapeutic candidates require additional validation to determine whether their effects are directly mediated through *ANLN*.

In summary, this study reveals the pivotal role of *ANLN* in lung cancer recurrence and lymph node metastasis from a multi-level and multi-scale perspective. Through integrative transcriptomic analyses, single-cell profiling, trajectory inference, and functional experiments, we demonstrate that *ANLN* is associated with aggressive epithelial programs and remodeling of the tumor microenvironment. Furthermore, network-based drug prediction combined with molecular docking analysis identified several candidate compounds potentially associated with *ANLN*-related molecular programs. Among these, Trifluridine and Monobenzone showed favorable docking interactions with *ANLN* and induced apoptosis in lung cancer cells in vitro. These results suggest that *ANLN*-associated molecular networks may represent a potentially actionable vulnerability in lung cancer. Although further mechanistic studies are required to determine whether these compounds directly target *ANLN*, our findings provide new insights into the regulatory mechanisms underlying lung cancer metastasis and highlight the potential translational value of *ANLN*-related pathways for therapeutic intervention.

## 5. Limitations

A major limitation of this study is that the majority of the analyses were conducted using publicly available bulk and single-cell transcriptomic datasets. Although multiple independent bulk cohorts and two independent single-cell datasets were included for survival analysis, differential expression analysis, and reproducibility validation, the overall sample size was constrained by the availability of eligible datasets in public repositories. Specifically, 425 tumor samples were used for survival analysis, 344 samples were included for recurrence- and metastasis-related differential expression analysis, and two independent single-cell RNA-seq datasets comprising 31 samples and approximately 230,000 cells in total were analyzed. Therefore, potential selection bias and residual heterogeneity across datasets cannot be fully excluded.

In addition, although functional experiments were performed to assess the effects of *ANLN* depletion and candidate drug treatment in lung cancer cells, the majority of the conclusions in this study remain based on computational and statistical analyses. The in vitro experiments primarily evaluated cellular phenotypes such as proliferation and apoptosis and did not further investigate the detailed molecular mechanisms underlying *ANLN*-mediated tumor progression. Moreover, the predicted small-molecule compounds were selected based on co-expression network-based enrichment analysis and molecular docking simulations. Although these compounds exhibited functional effects in cell-based assays, it remains unclear whether their antitumor activity is mediated through direct targeting of *ANLN*.

Furthermore, although single-cell transcriptomic data enabled cell-type-resolved analyses, the number of samples with detailed clinical annotations was limited, which may restrict the generalizability of the results across diverse patient populations. Future studies incorporating larger patient cohorts, in vivo models, and mechanistic experiments will be necessary to further substantiate the biological functions of *ANLN* and to clarify the therapeutic potential of *ANLN*-associated molecular pathways in lung cancer. In addition, a preliminary version of this study has been deposited as a preprint on the LangTaoSha Preprint Server (https://doi.org/10.65215/LTSpreprints.2026.02.20.000135).

## Figures and Tables

**Table 1:** Top predicted small-molecule compounds based on *ANLN* co-expression network analysis.

Index	Name	*p*-Value	Adjusted *p*-Value	Odds Ratio	Combined Score
1	Etoposide	6.199 × 10^−73^	1.166 × 10^−70^	823.72	136,955.57
2	Lucanthone	1.778 × 10^−116^	2.341 × 10^−113^	305.37	81,388.14
3	Testosterone	5.578 × 10^−109^	3.673 × 10^−106^	309.21	77,075.05
4	Trifluridine	6.885 × 10^−47^	6.045 × 10^−45^	594.12	63,149.96
5	Calcitriol	3.995 × 10^−92^	1.754 × 10^−89^	232.50	48,929.95
6	Monobenzone	5.372 × 10^−31^	2.721 × 10^−29^	585.12	40,782.09
7	Methotrexate	2.254 × 10^−52^	2.473 × 10^−50^	322.06	38,300.55
8	Ciclopirox	1.064 × 10^−57^	1.402 × 10^−55^	267.09	35,038.55
9	5109870	2.492 × 10^−50^	2.525 × 10^−48^	208.01	23,757.66
10	Dasatinib	1.051 × 10^−87^	3.459 × 10^−85^	100.07	20,041.32

**Table 2:** Molecular docking results of candidate compounds with *ANLN*.

Rank	Name	Model	Affinity (kcal/mol)	RMSD l.b. (Å)	RMSD u.b. (Å)
1	Etoposide	1	−7.8	0.000	0.000
		2	−7.7	0.110	1.960
		3	−7.2	14.349	18.414
		4	−7.2	33.843	37.917
		5	−7.2	4.307	10.247
2	Trifluridine	1	−7.5	0.000	0.000
		2	−7.5	2.317	3.425
		3	−7.4	2.292	9.638
		4	−7.3	24.057	27.128
		5	−7.3	2.746	3.893
3	Monobenzone	1	−7.0	0.000	0.000
		2	−6.9	12.375	14.556
		3	−6.8	36.178	38.904
		4	−6.7	5.395	10.635
		5	−6.7	12.042	14.051
4	5109870	1	−7.0	0.000	0.000
		2	−6.8	13.858	15.684
		3	−6.7	21.197	22.903
		4	−6.6	14.477	18.849
		5	−6.6	14.408	16.340
5	Ciclopirox	1	−6.8	0.000	0.000
		2	−6.4	2.459	8.434
		3	−6.3	27.841	30.232
		4	−6.1	26.679	28.745
		5	−6.0	29.705	34.062
6	Dasatinib	1	−6.3	0.000	0.000
		2	−6.1	30.025	33.767
		3	−6.0	30.317	34.060
		4	−6.0	1.038	2.492
		5	−5.8	15.872	16.817
7	Lucanthone	1	−6.2	0.000	0.000
		2	−6.2	26.893	27.820
		3	−6.1	22.177	25.828
		4	−6.1	1.483	2.218
		5	−6.0	2.099	3.713
8	Calcitriol	1	−5.7	0.000	0.000
		2	−5.3	24.918	26.608
		3	−5.2	12.755	14.074
		4	−5.1	31.881	34.302
		5	−4.9	32.669	34.776
9	Testosterone	1	−5.5	0.000	0.000
		2	−5.4	24.208	25.947
		3	−5.3	24.160	25.634
		4	−5.3	22.378	24.914
		5	−5.3	27.459	29.547
10	Methotrexate	1	−5.5	0.000	0.000
		2	−5.3	11.676	12.791
		3	−5.1	28.267	30.200
		4	−5.0	10.517	12.177
		5	−4.9	35.465	36.062

## Data Availability

All data used in this study are publicly available. Bulk RNA sequencing datasets used for survival and differential expression analyses were obtained from the Gene Expression Omnibus (GEO) database under the accession numbers: GSE157009 (https://www.ncbi.nlm.nih.gov/geo/query/acc.cgi?acc=GSE157009, accessed on 25 January 2026), GSE42127 (https://www.ncbi.nlm.nih.gov/geo/query/acc.cgi?acc=GSE42127, accessed on 25 January 2026), GSE41271 (https://www.ncbi.nlm.nih.gov/geo/query/acc.cgi?acc=GSE41271, accessed on 25 January 2026), and GSE166720 (https://www.ncbi.nlm.nih.gov/geo/query/acc.cgi?acc=GSE166720, accessed on 25 January 2026). Single-cell RNA sequencing datasets used for primary analysis and independent validation were also retrieved from GEO under the accession numbers: GSE277742 (https://www.ncbi.nlm.nih.gov/geo/query/acc.cgi?acc=GSE277742, accessed on 25 January 2026) and GSE189357 (https://www.ncbi.nlm.nih.gov/geo/query/acc.cgi?acc=GSE189357, accessed on 25 January 2026). Gene set enrichment analyses were performed using gene sets from the Molecular Signatures Database (MSigDB, version 7.5.1), including the Hallmark, Gene Ontology (GO), and KEGG collections (https://www.gsea-msigdb.org/gsea/msigdb/collections.jsp, accessed on 25 January 2026). Cell–cell communication analyses were conducted using curated ligand–receptor interaction databases implemented in CellChat (https://github.com/sqjin/CellChat, accessed on 25 January 2026). No newly generated datasets were produced in this study.

## Appendix A

### Validation of ANLN Knockout Effects on Proliferation and Apoptosis in H1975 Cells

Consistent results were observed in additional validation experiments performed in the H1975 cell line (Figure A1).
